# Fluorescence Tracking of Small Extracellular Vesicles In Vivo

**DOI:** 10.3390/pharmaceutics15092297

**Published:** 2023-09-08

**Authors:** Yanxia Chen, Yinghong Shi, Zhimin Tao

**Affiliations:** 1 Jiangsu Key Laboratory of Medical Science and Laboratory Medicine, Department of Laboratory Medicine, School of Medicine, Jiangsu University, Zhenjiang 212013, China; 2112113002@stmail.ujs.edu.cn; 2 Zhenjiang Key Laboratory of High Technology Research on Exosomes Foundation and Transformation Application, School of Medicine, Jiangsu University, Zhenjiang 212013, China

**Keywords:** small extracellular vesicles, fluorescence, near-infrared window, gastric cancer, small animal imaging

## Abstract

In this study, we employed organic and inorganic dyes that have fluorescence under visible or near-infrared light region to stain human umbilical cord (Huc) mesenchymal stem cell (MSC)-, HEK293T cell- and HGC cell-derived small extracellular vesicles (sEVs), and then tracked their fluorescence signals in human gastric cancer xenografted murine models. Several biological characteristics were examined and compared when different dye-stained sEVs in the same tumor model or the same dye-stained sEVs between different tumor models were applied, including sEVs circulation in the blood, biodistribution of sEVs in major organs, and time-dependent tumor accumulation of sEVs. The results demonstrated that distinct tumor accumulation features were presented by sEVs if labeled by different fluorescent dyes, while sEVs derived from different cell lines showed homologous blood circulation and tumor accumulation. To conclude, although fluorescence imaging remains a reliable way to trace sEVs, single staining of sEVs membrane should be obviated in future work when examining the biological fate of sEVs.

## 1. Introduction

Small extracellular vesicles (sEVs) with a membranous structure can be secreted by various types of human cells [1]. When first separated from ovine red blood cells in the 1970s, sEVs were considered to be plasma membrane-enclosed organelles of uniform size (50 nm) as a group of natural products from maturing reticulocytes [2]. Mounting evidence revealed that sEVs (e.g., exosomes) are subcellular vehicles with a size range of 30–200 nm, functioning as intercellular communicators and containing a variety of biomolecule cargoes, such as proteins, nucleic acids, and carbohydrates [1,3]. While inheriting main biological characteristics from parental cells, sEVs play an indispensable role in many physiological and pathological conditions, where their surface proteins may represent diagnostic biomarkers in various diseases and can be found in various body fluids (e.g., urine, blood) and tissues [4]. In parallel, as cell messengers, sEVs are the ideal delivery platform for human disease therapy, which have been widely applied in pharmaceutical and clinical research [5]. 

To better accomplish their delivery mission, EV-based carriers might be further modified for a desired pharmacokinetics and a preferred biodistribution. At the same time, many innate features of sEVs necessitate a judicious choice of specific cell origin for the biological system of interest. Since sEVs are produced from different biological sources with heterogenetic contents, especially their surface proteins, those aspects would greatly determine their efficacy of delivery and the ensuing therapeutics [1]. Moreover, sEVs unleashed from cells of the same type possess different sizes and shapes, which would influence their biological properties in vivo [6,7]. Thus, a combination of biological and physicochemical variables should be considered before certain sEVs are acquired or engineered for enhanced drug/biomolecular delivery. 

Diverse imaging strategies have been taken to track sEVs in vivo, including bioluminescence, fluorescence, radioactive labeling, and tomographic techniques [8]. Among them, fluorescence tacking is non-invasive, low-cost, and technique friendly. PKH dyes (named after their discoverer Paul Karl Horan) are the most common fluorescent dyes in the visible region that have been long used to stain small sEVs [9,10]. Although near-infrared (NIR) I fluorescence imaging (650–950 nm) provides a ‘transparency’ in the biological detections, recently upsurging developments in NIR-II fluorophores (emission at 1000–1700 nm) open a superlative biological tissue transparency window for biomedical research [11,12]. PbS QDs (emission at 1300 nm) have high quantum yield, higher resolution in biological imaging, deeper tissue penetration, and almost zero spontaneous fluorescence, making them an excellent choice for NIR-II region fluorescence imaging [13]. Therefore, in this study, we separated small sEVs derived from HucMSCs, HEK293T and HGC cells, and investigated their biological properties in human cancer cell-xenografted murine models, including pharmacokinetics, biodistribution and tumor accumulation, through the fluorescence tracking in the NIR-II windows to illustrate their imaging efficacy in comparison to traditional fluorescence imaging, with an aim to understand the in vivo properties of sEVs with different origins.

## 2. Materials and Methods

### 2.1. Chemicals and Cell Lines

All chemicals used in the study were purchased from Sigma-Aldrich, Ronkonkoma, NY, USA, unless otherwise specified. Human gastric cancer cell lines HGC-27 and Human Embryonic Kidney cell lines HEK293T were obtained from the cell bank of Chinese Academy of Sciences (Shanghai, China). The cell lines were cultured in high glucose Dulbecco’s Modified Eagle’s Medium (H-DMEM) (Bioind, Beit HaEmek, Israel) and supplemented with 10% fetal bovine serum (FBS) (ExCell Bio, Shanghai, China). Cells were incubated at 37 °C in humidified air with 5% CO_2_.

### 2.2. Isolation and Culture of HucMSCs

HucMSCs were isolated and characterized as previously reported [14]. Briefly, fresh human umbilical cord tissue was collected from the Affiliated Hospital of Jiangsu University from a volunteer donor and treated with phosphate buffered saline (PBS) (0.01 M, pH 7.4, Bioind, Israel), containing 100 U/mL penicillin and 100 mg/mL streptomycin (Biofil, Guangzhou, China). The tissues were washed and cut into small pieces (~1 mm^3^). Small tissues were cultured in minimum essential medium alpha (MEM-a) (Gibco, Billerica, MA, USA) containing 10% FBS (Gibco, USA) at 37 °C and 5% CO_2_. From the nineth day on, spindle-like cells were growing around the tissue block. As the cells expanded and formed a vortex or spiral shape, the tissue block was removed, and the adherent cells were trypsinized and placed in a cell culture flask or a large dish. As this step was completed, the first-generation cells were cultured. Here, in our study, the third- to eighth-generation HucMSCs under a good growth condition were used to extract HucMSC-derived sEVs.

### 2.3. Isolation and Characterization of sEVs

The culture supernatant of HucMSCs, HGC-27 and HEK293T was collected and centrifuged at 4 °C, 2 × 10^3^× *g* for 30 min to remove cell debris, and centrifuged at 4 °C, 1 × 10^4^× *g* for 30 min in an ultra-freezing centrifuge (Beckman, Brea, CA, USA) to remove impurity such as cell organelles. The upper layer liquid was transferred to the column of 100 kDa molecular-weight-cutoff (MWCO) ultrafiltration tubes (Millipore, Billerica, MA, USA), subject to centrifuge at 4 °C, 2 × 10^3^ *g* for 30 min to concentrate, followed by the removal of the bottommost concentrated liquid (dark red liquid) in the column. This process was repeated until there was no residual liquid in the column. The exosome extraction reagent (SBI, New York, NY, USA) was mixed with the concentrated solution at a volume ratio of 1:5 and precipitated at 4 °C for 12 h. Then, the precipitated concentrate was centrifuged at 4 °C, 2 × 10^3^× *g* for 30 min, and a white precipitate could be observed. An appropriate amount of PBS was added to dissolve the precipitate, forming the solutions of HucMSC-derived sEVs, HGC-27-derived sEVs and HEK293T-derived sEVs. The extracted sEVs solutions were sterilized through 0.22 μm filters (Millipore, USA), and then stored in a −80 °C refrigerator for later use. Protein quantification of these sEVs was performed using the BCA kit (CWBIO, Beijing, China). Furthermore, sEVs were characterized by the Western blotting, transmission electron microscopy (TEM) (Philips, Amsterdam, The Netherlands) and nanoparticle tracking analysis (NTA) (NanoSight, Malvern, UK). For Western blotting, equal amounts of proteins were separated by 12% sodium dodecyl sulfate-polyacrylamide gel electrophoresis (SDS-PAGE) and transferred to polyvinylidene fluoride (PVDF) membranes. After blocking with 5% skim milk for 1 h, membranes were incubated with primary antibodies and horseradish peroxidase (HRP)-conjugated secondary antibodies and detected using an enhanced chemiluminescence (ECL) substrate detection system (ImageQuant LAS 4000) (General Electric, Boston, MA, USA). The antibodies used in the experiment were CD63, CD81, HSP70, Calnexin (1:500), all purchased from Proteintech company (Proteintech, Rosamond, IL, USA). Primary antibodies were incubated at 4 °C overnight and HRP-conjugated goat anti-rabbit or goat anti-mouse secondary antibodies (1:2000) (CWBIO, China) were incubated at 37 °C for 2 h. For TEM characterization, sEVs were suspended and fixed in 100 mL of 2.5% glutaraldehyde overnight at 4 °C. Fixated sEVs were placed on carbon-coated copper grids and then stained with 2% uranyl acetate for 2 min. After washing, the samples were examined by TEM instrument (Tecnai G2 Spirit BioTWIN) (FEI, Pittsburgh, PA, USA).

### 2.4. Fluorescence Labeling of sEVs

A total of 6.4 mg/mL HucMSC-derived sEVs were incubated with the lipophilic fluorescence dye PKH26 (MKBio, Shanghai, China) at a concentration of 10 μM at 37 °C. Then, the mixture solution was transferred to the ultrafiltration tube with 100 kDa MWCO and centrifuged at 1500× *g* for 30 min to remove the unbound dye. The stained sEVs were resuspended in 200 μL PBS and passed through a 0.22 μm polyethersulfone (PES) filter (Millipore, USA) before use. Similarly, 6.4 mg/mL HucMSC-, HEK293T-, and HGC-derived sEVs were incubated with 0.5 mg/mL of lead sulfide (PbS) quantum dots (PbS QDs) (Nirmidas Biotech, Mountain View, CA, USA) at 37 °C, and the mixture was processed in an ultrasonic water bath for 3 min. Then, the mixture was transferred to the ultrafiltration tube with 100 kDa MWCO to be centrifuged under 1500× *g* for 30 min to remove the free PbS QDs, and finally the obtained solution was added to 200 μL PBS with a 0.22 μm PES filter, ready for use. Control groups with only the label were performed, and the labeling efficiency was calculated by the ratio of fluorescence intensity before and after labeling.

### 2.5. Dye Release Experiments

We studied the profiles of dye release in situ over time after sEVs were stained by PKH26 or PbS QDs individually. Specifically, the fluorescence of the newly stained PbS@HucMSCs-sEVs, PbS@HEK293T-sEVs, PbS@HGC-sEVs and PKH26@HucMSCs-sEVs was served as the initial fluorescence intensity. The same labeled sEVs were prepared to each time point. There were three duplicate samples in each group, and each sample was 200 μL. After being freshly labeled, sEVs were kept incubated at 37 °C. At different time points, i.e., 0, 0.5, 1, 2, 4, 8, 12, 24, and 48 h after the release started, the samples corresponding to each time point were taken and transferred to the ultrafiltration tube and centrifuged at 1500× *g* for 30 min to separate the dye stained sEVs and supernatant. The fluorescence of the supernatant was measured depending on the applied dye either by the Cytation 5 reader (BioTek Instruments, Winooski, VT, USA) or the DeepVision NIR-II in vivo imaging system (Nirmidas Biotech, USA) as used above. The fluorescence intensity of the obtained supernatant at each time point was normalized to the initial fluorescence intensity, obtaining the cumulative release of dyes from stained sEVs as a function of time. The release kinetics function$\;y=a\cdot \left(1-e^{-k\cdot t}\right)$ of the dyes was fitted according to the data, where a stands for the maximal release percentage and k denotes the releasing rate.

### 2.6. In Vivo Small Animal Experiments

Athymic nude mice (6-week-old female: male = 1:1) were purchased from Changzhou Cavins Laboratory Animal Co., Ltd. (Changzhou, China). Mice were subcutaneously injected with 200 μL of 1.0 × 10^7^/mL HGC-27 cells or 1.0 × 10^7^/mL HEK293T cells on both left and right flanks under anesthesia. Mice were closely monitored after the tumor cells were implanted, and animal experiments started when the tumor size grew to ~0.6 cm in diameter. At this point, sEVs labeled with different dyes were injected into the mice through tail-vein injection. The visible fluorescence imaging was obtained using the IVIS^®^ Spectrum In Vivo Imaging System (IVIS@ Lumina LT Series III) (PerkinElmer, Waltham, MA, USA), with the parameters preset to excitation wavelength of 535 nm and emission wavelength of 580 nm. The NIR-II fluorescence imaging was obtained using the DeepVision NIR-II in vivo imaging system. The 808 nm laser was applied for the excitation and the emission wavelength was 1300 nm. ImageJ software 1.8.0 (National Institutes of Health, Bethesda, MD, USA) was instrumented for further handling images. For a typical animal imaging experiment, the mice were anesthetized with 2.5% isoflurane and the sEVs were injected via the tail vein. Images were acquired at different time points (0.5, 1, 2, 4, 8, 12, 24, 48 h post injection or p.i.). The mice were sacrificed and ex vivo organs were imaged to obtain the biodistribution. All animal experiments were conducted following the guidelines of the Experimental Animal Administrative Committee of Jiangsu University.

### 2.7. Statistical Analysis 

Data analysis was performed using GraphPad Prism 8.0 software (GraphPad Software Inc., San Diego, CA, USA) and results were shown as mean ± standard deviation (SD). Differences between groups were calculated by multiple comparison test by Student’s t-test. *p* values <0.05 indicate a statistical significance.

## 3. Results

### 3.1. Characterizations of sEVs with Different Origins

**Figure 1:**
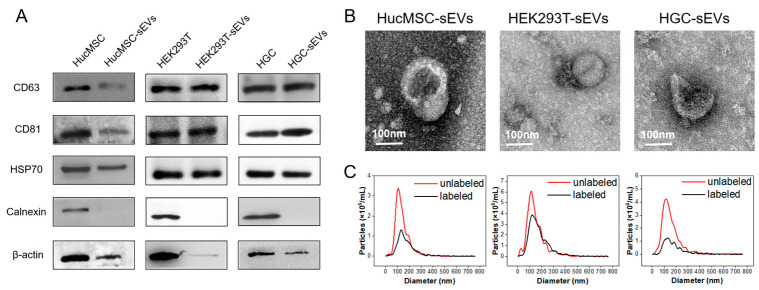
Characterizations of sEVs. (**A**) Western blot of different cells and their derived sEVs. (**B**) TEM images of sEVs showing sizes in a dimension of ~100 nm with a spherical or characteristic cup-like morphology. The scale bar indicates 100 nm. (**C**) Particle size distribution of sEVs detected by NTA, showing a peak size at ~130 nm.

The HucMSC-, HEK293T-, and HGC-derived sEVs were purified and collected as described, followed by the biological and physicochemical characterization. First, the Western blotting results confirmed that sEVs were enriched in many specific surface biomarkers, including β-actin, CD63, CD81 and HSP70, all inherited from their parental cells, but were depleted of calnexin (Figure 1A). TEM images (Figure 1B) illustrated that sEVs displayed a typical cup-shaped vesicle structure, with a dimension of ~100 nm. NTA measured the particle size distribution of sEVs with a peak value at ~130 nm (Figure 1C) and their surface charge was -25.0 ± 0.4 mV. The labeling efficiency of PKH26 and PbS QDs was about 60% and 53%, respectively. We also examined the particle size and zeta potential of sEVs after the staining of fluorescence dyes. Stained by lipophilic organic dye PKH26, the size of the PKH26-labeled HucMSCs-sEVs (i.e., PKH26@HucMSCs-sEVs) was ~154 nm and their surface charge was -26.4 ± 0.5 mV (results not shown), which was a bit larger in their hydrodynamic size with slightly more negative charge compared to the unstained one. Similarly, when PbS QDs were used to stain sEVs, the size of the formed PbS QDs@sEVs was ~149 nm and their zeta potential was -24.8 ± 0.1 mV. The swollen sizes of sEVs after staining procedures might correspond to the effective labeling of fluorescent tracers; whether PKH26 or PbS was stained, the positively charged carbocyanine dyes or amine-stabilized QD did not significantly add to the changes in the surface charges of sEVs. 

### 3.2. Dye Release Kinetics

**Figure 2:**
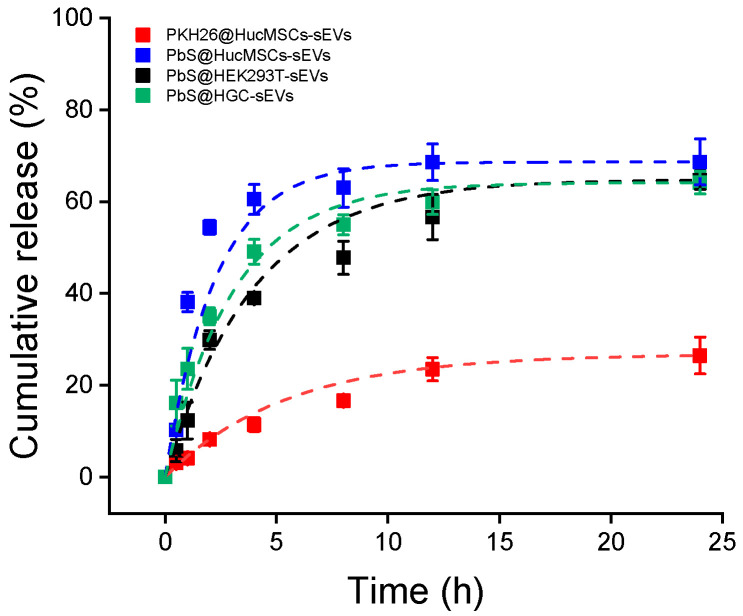
Dye leakage from stained sEVs. In situ releasing experiments of PKH26 from PKH26@sEVs or PbS QDs from PbS@sEVs. Each profile was fit into a function of release kinetics following $y=a\cdot \left(1-e^{-k\cdot t}\right)$, where a depicts the plateau of cumulative releasing per centage and k represents the releasing rate.

The dye release experiments were conducted in PBS solutions and results were shown in Figure 2. Clearly, PbS@sEVs demonstrated an initial burst of PbS release, where the cumulative release exceeded the half payload in the first 2 h. Since then, the releasing rate slowed down until the releasing plateau was reached. By fitting the data into a release kinetics function $y=a\cdot \left(1-e^{-k\cdot t}\right)$, where a stands for the maximal release percentage and k denotes the releasing rate, the values of a = 66.9% and k = 0.704 h^−1^ (R^2^ = 0.97) were obtained for the PbS@HucMSCs-sEVs releasing profile. In contrast, the PKH26@HucMSCs-sEVs displayed a sluggish unleashing of PKH26, and the fitting into the same function of release kinetics showed a = 27.4% and k = 0.141 h^−1^ (R^2^ = 0.99) for the PKH26@HucMSCs-sEVs releasing profile, confirming a significantly reduced releasing rate and a lessened releasing percentage in the timeframe examined up to 24 h (Table 1). 

### 3.3. In Vivo Pharmacokinetics, Tumor Accumulation and Biodistribution by Tracking sEVs in the Visible Fluorescence Region and the NIR-II Fluorescence Region

Human gastric cancer cells HGC-27 and Human Embryonic Kidney cells HEK293T were individually transplanted subcutaneously into the left and right flanks of the nude mice. After the xenografted tumors grew and progressed for 14 days, the tumors at both flanks in both models were 0.5–0.7 cm in diameter. After 16 h of staining, different dye-labeled sEVs were injected into nude mice bearing tumors through the tail vein at time = 0, and, at different time points p.i. as indicated, the mice were imaged by applying λ_ex_/λ_em_ = 535/580 nm in the visible fluorescence imaging window and λ_ex_/λ_em_ = 808/1300 nm in the NIR-II Fluorescence imaging window. At the same time, the mouse blood was collected, and the measured fluorescence intensity of the blood samples was plotted versus the collection time, which was fit into an exponential function with the decay constant obtained to calculate the circulation half-life (t_1/2_). In parallel, fluorescence signals reflected in the left and right tumor regions were recorded and normalized to the first fluorescence signal obtained and were plotted over time to display the dynamic tumor accumulation of sEVs. At 48 h p.i., the mice were sacrificed, followed by the harvest of major organs (e.g., heart, liver, spleen, etc.) and the measurement of their ex vivo fluorescence intensities. The fluorescence intensity of each tissue per tissue area was normalized to that of the liver, assessing the relative biodistribution of sEVs. All the measurements were calculated for all the systems (Table 2).

**Figure 3:**
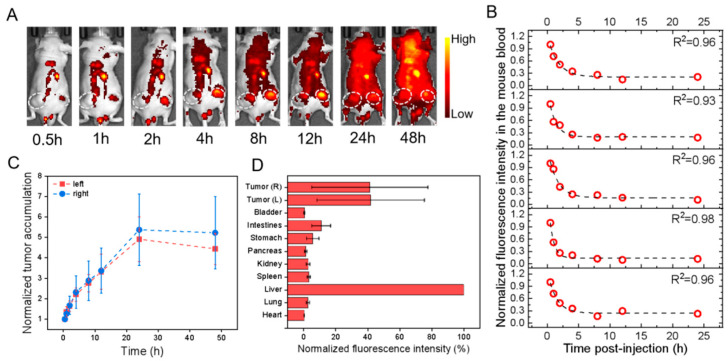
In vivo profiles of PKH26@HucMSCs-sEVs in HGC-27 cell-transplanted subcutaneous tumor models after tail-vein injection. All fluorescence images were taken upon excitation = 535 nm and emission = 580 nm. (**A**) Overlaid bright-field and fluorescence images of one representative mouse image with the tumor region monitored (dotted circle; only right flank shown) at different time points as indicated. (**B**) The fluorescence intensity of mouse blood was plotted versus different times p.i., which was fitted to an exponential function (indicated by the dashed line in each figure panel), so that the obtained exponential decay constant was converted into the circulation half-life time (t_1/2_) in the blood. (**C**) The fluorescence intensities of the left (red) and right (blue) tumors in the mice were monitored and plotted as a function of time (connected by dotted lines). Each fluorescence intensity at one specific time was normalized to that at the first time point and plotted versus time. (**D**) The mice were sacrificed at 48 h p.i., and the fluorescence intensities were examined for ex vivo organs as indicated with each normalized to that of the liver uptake, showing the relative fluorescence distribution in major organs.

**Figure 4:**
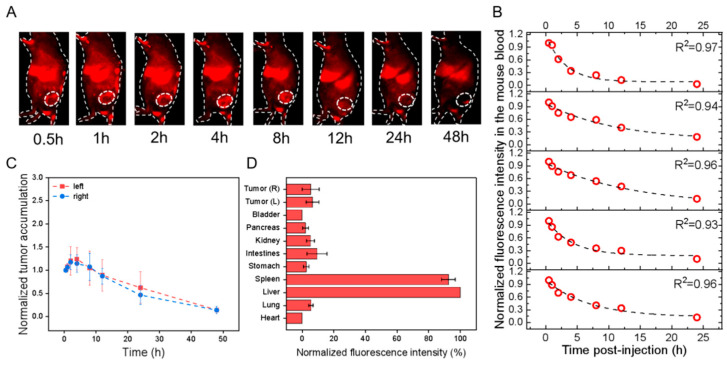
In vivo profiles of PbS@HucMSCs-sEVs in HGC-27 cell-transplanted subcutaneous tumor models after tail-vein injection. All fluorescence images were taken upon excitation = 808 nm and emission > 1000 nm. (**A**) Overlaid bright-field and fluorescence images of one representative mouse image with the tumor region monitored (dotted circle; only right flank shown) at different time points as indicated. (**B**) The fluorescence intensity of mouse blood was plotted versus different times p.i., which was fitted to an exponential function (indicated by the dashed line in each figure panel), so that the obtained exponential decay constant was converted into the circulation t1/2 in the blood. (**C**) The fluorescence intensities of the left (red) and right (blue) tumors in the mice were monitored and plotted as a function of time (connected by dotted lines). Each fluorescence intensity at one specific time was normalized to that at the first time point and plotted versus time. (**D**) The mice were sacrificed at 48 h p.i., and the fluorescence intensities were examined for ex vivo organs as indicated with each normalized to that of the liver uptake, showing the relative fluorescence distribution in major organs.

For the effect of the label, i.e., PKH26 versus PbS-QD, the results indicated that in the HGC-27 cells transplanted models, the circulation t_1/2_ of PKH26@HucMSCs-sEVs and PbS@HucMSCs-sEVs was nearly equal in both. The left and right tumor accumulation and distribution were nearly equal in each, but were much smaller for the PbS-QD-labeled than for PKH26-labeled sEVs (Figure 3C,D and Figure 4C,D). As a result, the PKH26@HucMSCs-sEVs and PbS@HucMSCs-sEVs reflected similar circulation time in the blood, despite completely distinctive tumor accumulation and tumor distribution at 48 h.

**Figure 5:**
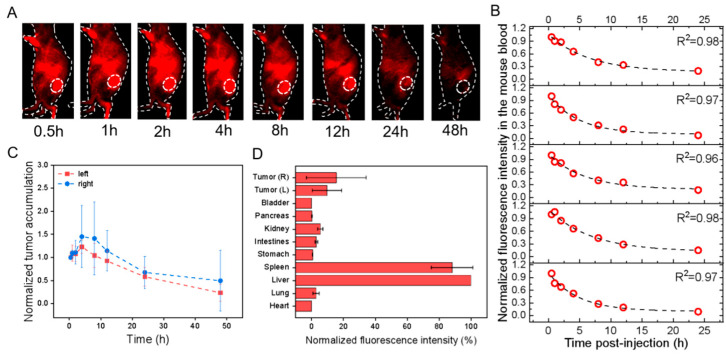
In vivo profiles of PbS@HucMSCs-sEVs in HEK293T cell-transplanted subcutaneous tumor models after tail-vein injection. All fluorescence images were taken upon excitation = 808 nm and emission > 1000 nm. (**A**) Overlaid bright-field and fluorescence images of one representative mouse image with the tumor region monitored (dotted circle; only right flank shown) at different time points as indicated. (**B**) The fluorescence intensity of mouse blood was plotted versus different times p.i., which was fitted to an exponential function (indicated by the dashed line in each figure panel), so that the obtained exponential decay constant was converted into the circulation t_1/2_ in the blood. (**C**) The fluorescence intensities of the left (red) and right (blue) tumors in the mice were monitored and plotted as a function of time (connected by dotted lines). Each fluorescence intensity at one specific time was normalized to that at the first time point and plotted versus time. (**D**) The mice were sacrificed at 48 h p.i., and the fluorescence intensities were examined for ex vivo organs as indicated with each normalized to that of liver uptake, showing the relative fluorescence distribution in major organs.

For the effect of cell lines, i.e., HGC-27 versus HEK293T, the results showed that the t_1/2_ of the PbS@HucMSCs-sEVs monitored in HGC-27 and HEK293T tumor-bearing nude mice was almost the same. The tumor accumulation in both models was nearly equal, but the tumor distribution in the HEK293T mouse model seemed to be higher than that in the HGC-27 mouse model (Figure 4C,D and Figure 5C,D). Therefore, the PbS@HucMSCs-sEVs showed similar circulation time and tumor accumulation in different tumor models. 

**Figure 6:**
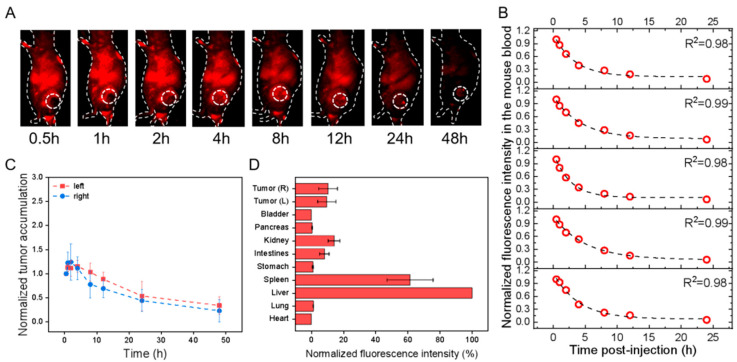
In vivo profiles of PbS@HEK293T-sEVs in HGC-27 cell-transplanted subcutaneous tumor models after tail-vein injection. All fluorescence images were taken upon excitation = 808 nm and emission > 1000 nm. (**A**) Overlaid bright-field and fluorescence images of one representative mouse image with the tumor region monitored (dotted circle; only right flank shown) at different time points as indicated. (**B**) The fluorescence intensity of mouse blood was plotted versus different times p.i., which was fitted to an exponential function (indicated by the dashed line in each figure panel), so that the obtained exponential decay constant was converted into the circulation half-life time (t_1/2_) in the blood. (**C**) The fluorescence intensities of the left (red) and right (blue) tumors in the mice were monitored and plotted as a function of time (connected by dotted lines). Each fluorescence intensity at one specific time was normalized to that at the first time point and plotted versus time. (**D**) The mice were sacrificed at 48 h p.i., and the fluorescence intensities were examined for ex vivo organs as indicated with each normalized to that of liver uptake, showing the relative fluorescence distribution in major organs.

**Figure 7:**
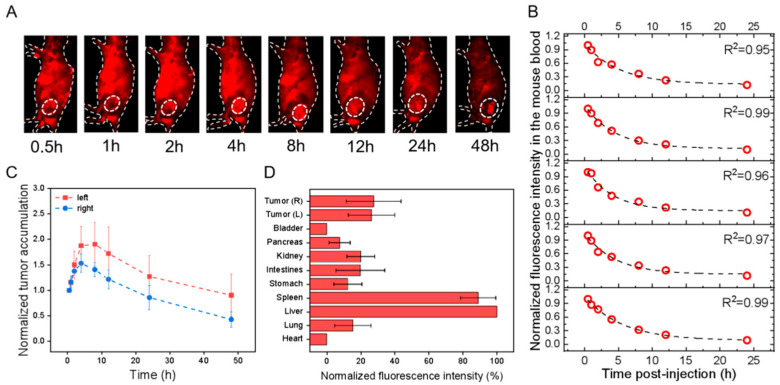
In vivo profiles of PbS@HGC-sEVs in HGC-27 cell-transplanted subcutaneous tumor models after tail-vein injection. All fluorescence images were taken upon excitation = 808 nm and emission > 1000 nm. (**A**) Overlaid bright-field and fluorescence images of one representative mouse image with the tumor region monitored (dotted circle; only right flank shown) at different time points as indicated. (**B**) The fluorescence intensity of mouse blood was plotted versus different times p.i., which was fitted to an exponential function (indicated by the dashed line in each figure panel), so that the obtained exponential decay constant was converted into the circulation t_1/2_ in the blood. (**C**) The fluorescence intensities of the left (red) and right (blue) tumors in the mice were monitored and plotted as a function of time (connected by dotted lines). Each fluorescence intensity at one specific time was normalized to that at the first time point and plotted versus time. (**D**) The mice were sacrificed at 48 h p.i., and the fluorescence intensities were examined for ex vivo organs as indicated with each normalized to that of liver uptake, showing the relative fluorescence distribution in major organs.

For the effect of sEVs origin, i.e., HucMSC-sEVs, HEK293T-sEVs and HGC-sEVs, the results showed that the t_1/2_ of the PbS@HucMSCs-sEVs, PbS@HEK293T-sEVs and PbS@HGC-sEVs was nearly the same. However, the tumor accumulation and distribution of the PbS@HGC-sEVs were slightly higher than those of the other two sEVs (Figure 4C,D, Figure 6C,D and Figure 7C,D). The results indicated that the sEVs derived from different cell sources exhibited similar circulation time in the blood and a little different of the accumulation/distribution in tumors.

## 4. Discussion

Compared to visible imaging, NIR-II fluorescence imaging has demonstrated many advantages, such as much reduced light scattering, diminished autofluorescence background, and enhanced tissue penetration [15,16]. When NIR-II fluorescence technique was devised at its infancy, only a handful of imaging dyes were available [17,18]. To date, a variety of novel NIR-II fluorophores have been developed to actively fuel the research field, including inorganic composites typified by rare-earth doped nanomaterials, quantum dots and carbon nanotubes, and organic compounds such as small-molecule fluorescence dyes and semiconducting polymer nanoparticles [12,19,20,21,22]. Currently, biosafe, biocompatible and biodegradable NIR-II fluorophores with non-immunogenicity are keenly sought for their brightness in clinical translation. In our study, PbS QDs were used for NIR-II fluorescence imaging. The results showed that, compared to imaging in the visible fluorescence region, NIR-II imaging had lower light scattering and higher signal-to-noise ratio, which could clearly reflect excellent fluorescence signals of the entire animal, mouse blood, and other external organs.

As a natural biological vesicle, sEVs have been widely used as drug delivery carriers [23]. And the pharmacokinetics of sEVs has been understudied in recent years. Many reported results did not agree with each other, mostly due to different methodologies applied and different sEVs of various cell origins. Using globular sEVs separated from cell cultures of five murine cell lines, with a similar diameter of ~100 nm, researchers found out that irrespective of separation methods, murine cell sources, CD81 expression levels, or bioluminescence/fluorescence-labeled vesicle tracking, sEVs exhibited a half-life of ~2–4 min [24,25,26,27]. Similarly, Expi293F human cell-derived sEVs with sizes of 126–154 nm were found to be cleared out of the tumor-bearing BALB/c mice within 10 min, and surface variation in sEVs modified their physiological destinations [28].

Moreover, human umbilical cord mesenchymal stromal cell-derived sEVs, with the hydrodynamic size of ~171 nm (polydispersity index = 0.43) and the zeta potential of ~-16 mV, were found to accumulate within the subcutaneously xenografted mouse osteosarcoma tumor of immunocompromised nude mice even 24–48 h p.i. via the tail vein [29]. This result suggested a prolonged circulation time of the applied sEVs; although, the exact pharmacokinetics was unexamined in the report. Similarly, human HEK293T cell-derived sEVs (a mixture of extracellular vesicles with a size range of 60–200 nm) were intravenously injected into human glioma cell line Gli36-xenografted athymic nude mice, showing a distribution half-life of ~20 min, an elimination half-life of > 3 h, and a tumor accumulation duration from 1 h to 6 h p.i. [30]. Therefore, the blood circulation of sEVs can be adjusted by employing sEVs from various sources or engineering sEVs to impart them with tunable surface properties [31]. In this study, we evaluated the pharmacokinetics of sEVs under three different conditions, namely the effect of dyes, the effect of tumor models, and the effect of sEVs origins. The research results showed that the circulation time of sEVs was similar, both of about 4 h. This may be related to the morphology and the size of sEVs after labeling. The results may also indicate that our method of labeling sEVs with different dyes to detect their circulation time in vivo was feasible and could better reflect the situation of sEVs in vivo. 

Biodistribution profiles could be altered when sEVs are obtained from different cell sources (with varying physical dimension, inner composition, and surface proteins), administrated via different injection routes, or given with different dosages [32,33,34]. As a result, distinctive patterns of biodistribution were observed in C57BL/6 mice, where large exosomes showed higher lymph node tropism and exomeres exhibited more liver uptake compared to small exosomes [35]. In this study, we traced sEVs from different labels and origins. The results indicated that the size of the labeled sEVs was approximately 150 nm and the surface charge was about ~-26 mV, which was not significantly different from the unlabeled sEVs. We found that when the two dyes with different properties were used for labeling, most of the fluorescence signals ultimately concentrated in the liver, but the fluorescence of other organs varied. When PKH26 was used to label sEVs, the fluorescence at the tumors was higher than that of other organs. However, when the PbS QDs were used to label sEVs, there was also a large amount of fluorescence aggregation in the spleen, while the fluorescence at the tumors was relatively weak. These conflicting results may suggest that organic and inorganic dyes have different binding capabilities or mechanisms to sEVs and once incorporated into sEVs, their releasing profiles under physiological or pathological conditions could greatly vary. It is known that PKH26 inserts their long aliphatic carbon tails into the exosomal membrane during staining [36], whereas staining using the positively charged amine-modified QD may adsorb or deposit QDs of heavy metals onto the external membrane of sEVs with a negative charge. For this reason, tracking sEVs in vivo by following the individual fluorescence reporter could render complicated results [10,36,37].

Tumor accumulation could be augmented by functionalizing sEVs surface with targeting moiety or taking advantage of sEVs with certain origin for their homing tropism. For instance, the tripeptide Arg-Gly-Asp (RGD) was conjugated to the external surface of complex sEVs for targeting integrin-rich tumors in a human lung cancer cell line xenografted murine model, showing a 1.2-time increase in tumor distribution compared to non-targeting particles [38]. Moreover, more efficient uptakes of sEVs (1.4–2 times higher) by parental cancer cells were observed than those by cancer cells of differing types in subcutaneous rodent models [39]. Recently, this cancer homing tropism was generalized for tumor-derived sEVs when targeting any type of tumor malignancy, even at the early stage of neoplastic transformation, which could be cross-cancer and cross-species [40,41]. In this study, sEVs with different dye labels and origins were compared. The results showed that the differences in dye properties led to distinct trends in their accumulation in tumors. The tumor accumulation of sEVs labeled with the lipophilic dye PKH26 gradually increased in the tumor and eventually stabilized or slightly decreased. However, for sEVs labeled with PbS QDs, regardless of the tumor models, their accumulation in the tumor showed a trend of first increasing and then decreasing, with peaks concentrated around 4–8 h. For sEVs from different origins, their tumor accumulation also varied slightly. Compared to the other two origins, sEVs derived from HGC had more tumor accumulation, with a peak of approximately 8 h. Secondly, there were sEVs derived from HucMSC, while sEVs derived from HEK293T had a weaker accumulation in the tumor. The reason for this result may be the diversity of integrin expression on the surface of sEVs, while sEVs derived from parental cancer cells had a cell-derived tendency, resulting in more accumulation in tumors [39].

Among all fluorophores employed in in vitro and in vivo studies, organic fluorescent dyes and quantum dots have been widely applied in pre-clinical animal models [42,43,44]. In this study, we adopted PKH26 and amine-modified PbS QDs to stain HucMSC-derived sEVs for in vivo fluorescence imaging in human cancer xenografted murine model. HucMSC-sEVs owned a hydrodynamic size of ~130 nm with zeta potential of ~−25 mV, while the PKH26 or PbS staining did not add substantial changes in their size and surface charge. For the same dye staining the HucMSC-sEVs, HEK293T-sEVs and HGC-sEVs in HGC-27 xenografted murine models, the biological characters, including the circulation half-life time and the biodistribution pattern, were similar. However, for the same gastric tumor model but detected through different fluorescence signals, the tumor accumulation of sEVs showed distinctiveness. 

Further technical improvement can be considered to stain and track sEVs in vivo, such as immunolabeling by tagging fluorescent dyes to specific antibodies against exosome surface protein [45], chemical conjugation of fluorescent dyes to external surface of sEVs via click chemistry [46], or employment of multiplexed [47] or multimodal reagents through luminal labeling of exosomal internal space [48].

## 5. Conclusions

In our study, within the same human cancer murine model, sEVs labeled by different fluorescent dyes showed similar circulation time and completely distinctive tumor accumulation features, revealing the differences exhibited by dyes with different properties when tracking sEVs, while no significant difference was seen in the metabolic and circulatory patterns of sEVs from different origins in the same cancer murine model.

## Figures and Tables

**Table 1:** The fitting parameter values of the plateau of cumulative releasing percentage and releasing rate for the dye release kinetics.

Group	a Value	k Value	R^2^
PKH26@HucMSCs-sEVs	27.4	0.141	0.99
PbS@HucMSCs-sEVs	66.9	0.704	0.97
PbS@HEK293T-sEVs	63.1	0.248	0.97
PbS@HGC-sEVs	60.4	0.428	0.97

**Table 2:** The values of the circulation half-life (t_1/2_), tumor accumulation and distribution for all the systems.

Tumor	sEVs	t_1/2_ (h)	Left Tumor Accumulation (Fold)	Right Tumor Accumulation (Fold)	Left Tumor Distribution (%)	Right Tumor Distribution (%)
HGC	PKH26@HucMSCs-sEVs	3.4 ± 0.7	4.4 ± 0.8	5.2 ± 1.8	41.9 ± 33.5	41.4 ± 36.3
HGC	PbS@HucMSCs-sEVs	3.9 ± 0.2	0.2 ± 0.1	0.1 ± 0.1	6.7 ± 4.0	5.5 ± 5.3
HEK293T	PbS@HucMSCs-sEVs	4.4 ± 0.1	0.2 ± 0.2	0.5 ± 0.6	9.8 ± 9.2	15.6 ± 18.7
HGC	PbS@HEK293T-sEVs	4.6 ± 0.4	0.3 ± 0.2	0.2 ± 0.2	9.6 ± 5.7	10.4 ± 5.8
HGC	PbS@HGC-sEVs	4.3 ± 0.2	0.9 ± 0.4	0.4 ± 0.1	26.3 ± 13.6	27.6 ± 16.1

## Data Availability

The raw data supporting the conclusions of this article will be made available by the authors, without undue reservation.
